# HHG at the Carbon K-Edge Directly Driven by SRS Red-Shifted Pulses from an Ytterbium Amplifier

**DOI:** 10.1021/acsphotonics.2c01021

**Published:** 2022-12-29

**Authors:** Martin Dorner-Kirchner, Valentina Shumakova, Giulio Coccia, Edgar Kaksis, Bruno E. Schmidt, Vladimir Pervak, Audrius Pugzlys, Andrius Baltuška, Markus Kitzler-Zeiler, Paolo Antonio Carpeggiani

**Affiliations:** † Photonics Institute, Technische Universität Wien, A-1040 Vienna, Austria; ‡ Christian Doppler Laboratory for Mid-IR Spectroscopy and Semiconductor Optics, University of Vienna, A-1090 Vienna, Austria; § Istituto di Fotonica e Nanotecnologie-Consiglio Nazionale delle Ricerche (IFN-CNR) and Dipartimento di Fisica-Politecnico di Milano, Piazza Leonardo da Vinci 32, Milano 20133, Italy; ∥ few-Cycle Inc., 1650 Blvd. Lionel Boulet, J3X 1P7, Varennes, QC Canada; ⊥ Ludwig-Maximilians-Universität München, Department of Physics, Am Coulombwall 1, 85748 Garching, Germany; # UltraFast Innovations GmbH, Am Coulombwall 1, 85748 Garching, Germany; ○ Center for Physical Sciences and Technology, Savanoriu Ave. 231, LT-02300, Vilnius, Lithuania

**Keywords:** high harmonic generation, carbon K-edge, water window, stimulated Raman scattering, ytterbium amplifier

## Abstract

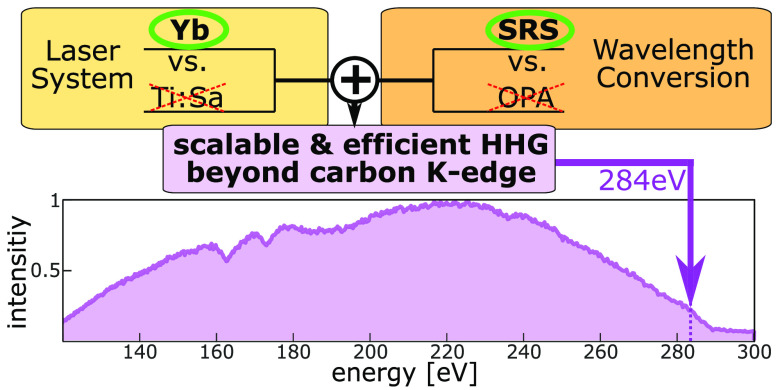

In this work, we introduce a simplified approach to efficiently extend the high harmonic generation (HHG) cutoff in gases without the need for laser frequency conversion via parametric processes. Instead, we employ postcompression and red-shifting of a Yb:CaF_2_ laser via stimulated Raman scattering (SRS) in a nitrogen-filled stretched hollow core fiber. This driving scheme circumvents the low-efficiency window of parametric amplifiers in the 1100–1300 nm range. We demonstrate this approach being suitable for upscaling the power of a driver with an optimal wavelength for HHG in the highly desirable XUV range between 200 and 300 eV, up to the carbon K-edge. Due to the combination of power scalability of a low quantum defect ytterbium-based laser system with the high conversion efficiency of the SRS technique, we expect a significant increase in the generated photon flux in comparison with established platforms for HHG in the water window. We also compare HHG driven by the SRS scheme with the conventional self-phase modulation (SPM) scheme.

## Introduction

### High Harmonic Generation

High harmonic generation (HHG) in gases, driven by near-infrared (NIR) femtosecond laser pulses, has been widely used for time-resolved investigations of ultrafast electronic and molecular dynamics with a variety of techniques. More recently, the generation of soft X-ray pulses in the water-window spectral region^1,2^ has been used for time-resolved investigations of molecular dynamics by transient absorption at the near edge (K or L edge) of the constituting elements of organic molecules in the gas^3^ or liquid phase.^4^ The transparency of water in this spectral region also enables the observation of molecules in the aqueous solution,^5^ often the most natural environment of biochemical compounds. Also, because absorption edges of several key elements (K-edges of carbon at 284 eV, nitrogen at 410 eV, and oxygen at 540 eV) of organic and biochemical important molecules are situated in the water window, it is particularly interesting for near-edge X-ray absorption fine structure (NEXAFS) based techniques.^3,6,7^ Among the elements that exhibit absorption edges in the water window, especially carbon, due to its ability to form a variety of stable bonds (single, double, and triple, and structures with delocalized electrons) with many elements, including itself, provides the core structure for organic chemistry and therefore a vast research target. To give a few examples, the elemental specificity and chemical sensitivity of NEXAFS enabled the time-resolved observation of ultrafast ring-opening,^8,9^ intersystem crossing,^10^ and bond dissociation.^11,12^

In general, HHG is achieved by focusing intense, NIR femtosecond laser pulses in a noble gas, where the strongly nonlinear light–matter interaction results in the emission of light at much higher frequencies (extreme ultraviolet (XUV) or soft X-ray) as compared to the one of the drivers (NIR). This process is well understood both at the microscopic level of the single atom response^13,14^ and at the macroscopic level through the phase-matching conditions.^15^ The generated spectrum features a broadband plateau that quickly drops at the so-called cutoff energy. When targeting HHG in a specific spectral region, the main scaling laws to be taken into account are the dependence of the cutoff energy *E*_cutoff_ ∝ *I*λ^2^^13,14^ and of the harmonic photon flux Φ ∝ λ^–5^ – λ^–6^^16,17^ on the driver wavelength. The highest HHG conversion efficiency is therefore obtained at the shortest NIR driver wavelength for which HHG just reaches the targeted XUV region. This NIR driver wavelength can be considered the optimal driver wavlength λ_OD_. Indeed, with driver wavelengths λ_D_ < λ_OD_, the HHG process would not reach the targeted region, while with λ_D_ > λ_OD_, the target would be reached, but with a reduced photon flux. The two opposite dependencies considered above are derived at the level of single-atom response, while the macroscopic, observable harmonic spectra result from the phase-matching conditions. An exhaustive theoretical analysis of phase-matching conditions for driving wavelengths between 800 nm and 10 μm, supported by experimental data at 800 nm and at 1300 nm, has already been reported.^18^ The role and the possible combinations of driver wavelength, pulse duration, the type and pressure of generating medium are considered for the calculation of the cutoff limit of phase-matched harmonics. Regarding the photon flux that can be achieved by phase-matched harmonics with different combinations of driver wavelength and generating medium, a recent experimental and theoretical investigation from our group^19^ showed that HHG in helium with λ_D,He_ = 1030 nm yield higher photon flux above 170 eV, as compared to neon, with λ_D,Ne_ = 1500 nm, with an absolute value of flux Φ = 2 × 10^9^ photons/s/1%BW at 200 eV. In that case, and as we also confirm in this work under similar conditions, the cutoff energy was about 220 eV. As reported in Table 1, several works show cutoff energies above 300 eV with a driver wavelength of 1300 nm.^7,8,18^ Thus, in the specific case of HHG targeting the 220–300 eV range (covering, among others, the sulfur L-edge and the carbon K-edge), the optimal driving wavelength would be in the 1100–1300 nm range with helium as the generating medium.

**Table 1:** Comparison of Laser Systems Capable of Driving HHG in the Water Window Spectral Region^6,8,18,32−42^ with Recent Approaches Using SRS-Based Red-Shift and Postcompression^26,27^ and This Work

HHG		driving laser parameters						
cutoff (eV)	gas	system	wavelength (μm)	pulse duration (fs)	rep. rate (Hz)	pulse energy (mJ)	power (mW)	ref
330	He	Ti:Sa +OP(CP)A	1.30	35	10	5.50	55	(19)
300	He		1.32	50	1000	2.80	2800	(8)^a^
400	Ne		1.50	50	1000	1.60	1600	(33)
270	Ne		1.55	45	10	100.00	1000	(34)^a^
340	He							
320	Ne		1.60	9	1000	0.55	550	(35)
300	Ne		1.60	35	10	2.20	22	(36)
450	He					4.50	45	
350	Ne		1.80	50	1000	2.50	2500	(6)^a^
375	Ne		1.80	8	1000	0.70	700	(37)^a^
543	He		1.80	30	100	7.85	785	(38)
390	Ne		1.85	12	1000	0.40	400	(39)
350	Ne		1.85	12	1000	0.40	400	(40)
500	He							
395	Ne		2.00	40	10	2.40	24	(41)
530	He							
								
450	Ne	Yb+OPCPA	2.10	32	1000	1.35	1350	(42)
1600	He		3.90	80	20	10.00	200	(43)
								
80	Ar	Yb+SRS	1.20	<10	50000	0.245	12250	(26)
80	Ar	Ti:Sa + SRS	0.940	10.8	100	2.42	242	(27)
								
165	Ne	Yb+SPM	1.03	18.5	500	9.00	4500	this work
220	He		1.03	18.5	500	9.00	4500	
200	Ne	Yb+SRS	1.23	22.0	500	8.00	4000	
290	He		1.23	22.0	500	8.00	4000	

a These references also include the successful application to absorption spectroscopy.

### Harmonic Flux and Power Scaling of the Driver

Given the intrinsic extremely low conversion efficiency from the NIR driver into the soft X-ray spectral region via HHG in gases, the main limitation of this technique lies in the difficulty to increase the photon flux Φ, defined as the number of generated photons per laser pulse times the pulse repetition rate. Such an increase would be extremely beneficial, as it would allow to study more complex molecular samples and the quantitative determination of the branching ratios in the transient products of the reaction, as well as the study of molecular dynamics for samples in liquid solutions rather than in the gas phase, which for most samples is quite an artificial environment.

An ideal driver for HHG should deliver an energetic (several mJ), short (tens of fs) pulse with a tunable wavelength in the NIR and at high repetition rate (kHz). The requirements of high peak power pulses and high repetition rate narrow down the pool of possible laser sources to titanium sapphire (Ti:Sa; 800 nm, 20–50 fs) and ytterbium amplifiers (1030 nm, 200 fs). However, to achieve cutoff energies beyond 220 eV, an intermediate step is necessary for converting the laser fundamental into a longer wavelength for the HHG driver.

A typical way to obtain tunable few-mJ pulses with a duration of several tens of fs is by optical parametric amplification (OPA). The conversion efficiency and spectral bandwidth of OPA is limited by the properties of nonlinear crystals, such as the nonlinear coefficient *d*_eff_, group velocity mismatch between interacting pulses, crystal length, and optical damage threshold.^20−22^ At moderate pump energies of several mJ the conversion efficiency of pump to both signal and idler waves combined, when close to the doubled pump wavelength, can be as high as 50%.^23^ However, it drops fast with detuning from this degeneracy point. Following Manley–Rowe relations,^21^ the typical conversion efficiency of ∼10–25% and 2–10% can be achieved solely in signal and idler pulses correspondingly. Working at the high conversion efficiency regime and therefore at high intensities leads to the degradation of the beam due to a parametric back conversion at the center of the beam and might affect the pulse quality. Keeping a high beam fidelity and scaling to higher energies requires to lower the intensity and increase the size of the beam. Often, this energy scaling is restricted by available crystal apertures, their spatial homogeneity, onset of small-scale self-focusing, and subsequent nucleation of the beam. However, it is still possible by using the optical parametric chirped amplification (OPCPA) approach.^24,25^ OPCPA systems allow to generate ultrashort pulses with tens of mJ of energy, however, require complex dispersion management and therefore are limited in wavelength-tunability.

An alternative is the recently demonstrated possibility by stimulated Raman scattering (SRS) to induce an asymmetric spectral broadening toward the longer wavelengths in laser pulses from both Ti:Sa and ytterbium based lasers by propagation in a long, stretched, hollow core fiber (HCF) filled with molecular gases. Here, the new spectral components at longer wavelengths can contain more than 80% of the pulse energy.^26−28^ This effect can be seen as a spectral broadening combined with moderate red-shift, and it is indeed suitable for the generation of compressed pulses as in the case of self-phase modulation (SPM), but red-shifted in the vicinity of the laser wavelength.

**Figure 1:**
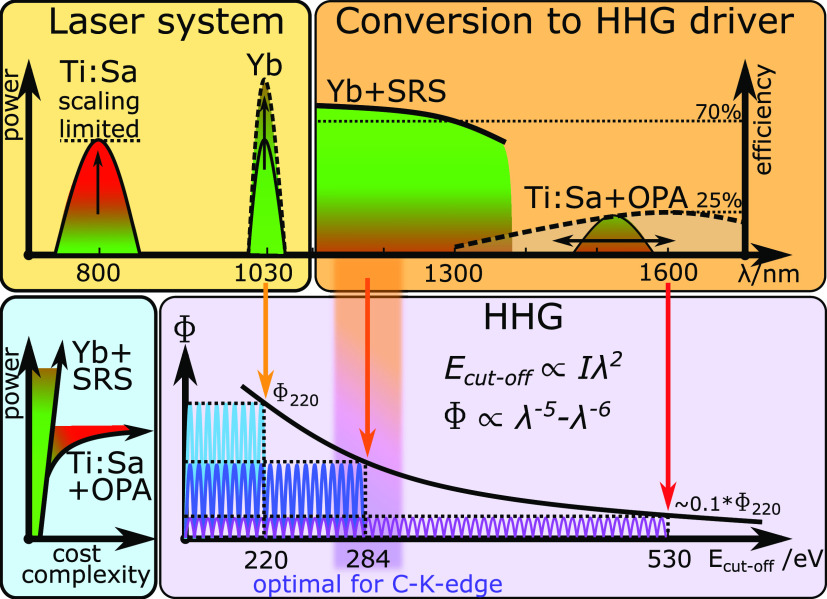
Schematic concept of our approach to increase the photon flux Φ at the carbon K-edge. Top left, Yb lasers are more suitable for power scaling as compared to Ti:Sa lasers. Top right, the generation of pulses in the 1200–1300 nm range is more efficient with Yb+SRS as compared to Ti:Sa+OPA. Bottom left, a schematic of power scaling vs cost and complexity for the two systems. Bottom right, the dependence, as determined by the scaling laws of HHG, of the achievable cutoff energy *E*_cutoff_ and photon flux Φ as a function of the driving laser wavelengths λ given by the upper plot. The values for *E*_cutoff_ are estimates based on the 220 eV cutoff energy achieved by direct driving with our ytterbium-doped calcium fluoride crystal (Yb:CaF_2_) laser system at 1030 nm.

In Figure 1 we show a schematic concept of our approach to increase the achievable photon flux Φ at the carbon K-edge by tackling this task from each of the three steps involved: the efficiency of conversion of the HHG process, the efficiency of conversion from the laser fundamental into the driver wavelength, and the power scaling of the laser source. The efficiency of conversion of the HHG process is improved by properly choosing the optimal wavelength for the driver, i.e. the shortest one which still allows to cover the desired cutoff energy. Then we determine the combination of laser system and wavelength conversion process that, together, can access this optimal wavelength with the highest efficiency. A further increase of the flux in the soft X-ray spectrum can then be achieved by the power scaling of the laser source. Following this concept, we will discuss below that the proposed scheme of ytterbium laser system in combination with SRS based wavelength conversion represents the ideal platform for scaling the photon flux at the carbon K-edge.

Ti:Sa laser and OPA are well established and reliable technologies, which also means that their improvement in terms of power scaling and efficiency can be only incremental. In details, Ti:Sa have been, until recently, pumped indirectly: typically, a diode laser at 808 nm pumps a Nd:YAG laser, which emits light at 1064 nm, which is subsequently frequency doubled to 532 nm to pump the Ti:Sa. In the perspective of power scaling, the efficiency, limitations and complexity of each of these steps must be accounted for. Even though nowadays Ti:Sa laser can be directly pumped by diodes in the blue (450 nm)^29^ or green (520 nm)^30^ wavelength range to produce laser pulses around 800 nm, their power scalability suffers fundamentally from the higher quantum defect when compared with ytterbium based laser systems that are directly diode pumped around 970 nm to deliver laser pulses at 1030 nm. Conversely, due to their smaller quantum defect, ytterbium based gain media represent the best option for power scalability of ultrashort laser pulses. On the other hand, their smaller gain bandwidth limits the directly achievable pulse duration, as compared to Ti:Sa. Given the possibility to postcompress the pulses from ytterbium amplifiers by SPM in a HCF to tens of fs or below,^31^ the longer wavelength is particularly advantageous when targeting HHG in the 100–200 eV region,^19^ but insufficient to reach the carbon K-edge. For this, longer driving wavelengths are required, as can be provided by SRS in a HCF. It has been demonstrated that this SRS technique is suitable also for driving HHG,^26,27^ but these first investigations showed only the possibility to reach 80 eV when focusing the red-shifted and compressed pulses in argon. The advantage expected from SRS, but not confirmed prior to our work, is the possibility to efficiently extend the cutoff energy of generated harmonics due to the red-shift in the fundamental wavelength beyond the limits of pulses compressed by SPM. We show that the combination of the Yb:CaF_2_ laser with SRS in HCF allows us to extend the cutoff of HHG in helium from 220 to 290 eV, thus reaching the carbon K-edge, with an optimal driving wavelength without additional conversion losses and complexity from an OPA stage.

A comparison of several laser systems capable of driving HHG in the water window with recent attempts of driving HHG with pulses produced via SRS, and our work is shown in Table 1. Recently, considerable effort went into increasing the pulse energies in the water window spectral region.^33^ Considering the requirements of pulse energy, repetition rate, and pulse duration, the preferred platform for near edge transient absorption experiment has been Ti:Sa lasers in combination with OPA,^1−3^ and experiments so far relied on driving NIR wavelengths above 1300 nm. The choice of this driving wavelength is dictated by the range of efficient conversions of OPA rather than by the optimum for the HHG process. Of the shown platforms, only refs (8) and (18) employ a driver wavelength close to the optimal range for targeting the carbon K-edge due to the unfavorable efficiency of OPA systems toward the pump wavelength. The longer driving wavelength for HHG also covers the carbon K-edge, but for a given HHG gas at a reduced conversion efficiency.^6,32−42^ In this work, we demonstrate the successful application of SRS red-shifted pulses as a driver to generate phase matched harmonics with a cutoff extended well beyond the limit of laser pulses at the unshifted fundamental laser wavelength produced by SPM. Most importantly, we demonstrate the extension of the cutoff up to the carbon K-edge at 284 eV with HHG driven in helium. In terms of laser parameters of the drivers for HHG reaching the carbon K-edge, Table 1 shows that the proposed approach already features the highest average power, as well as the shortest duration among the systems with multi-mJ pulses.

## Experimental Setup

**Figure 2:**
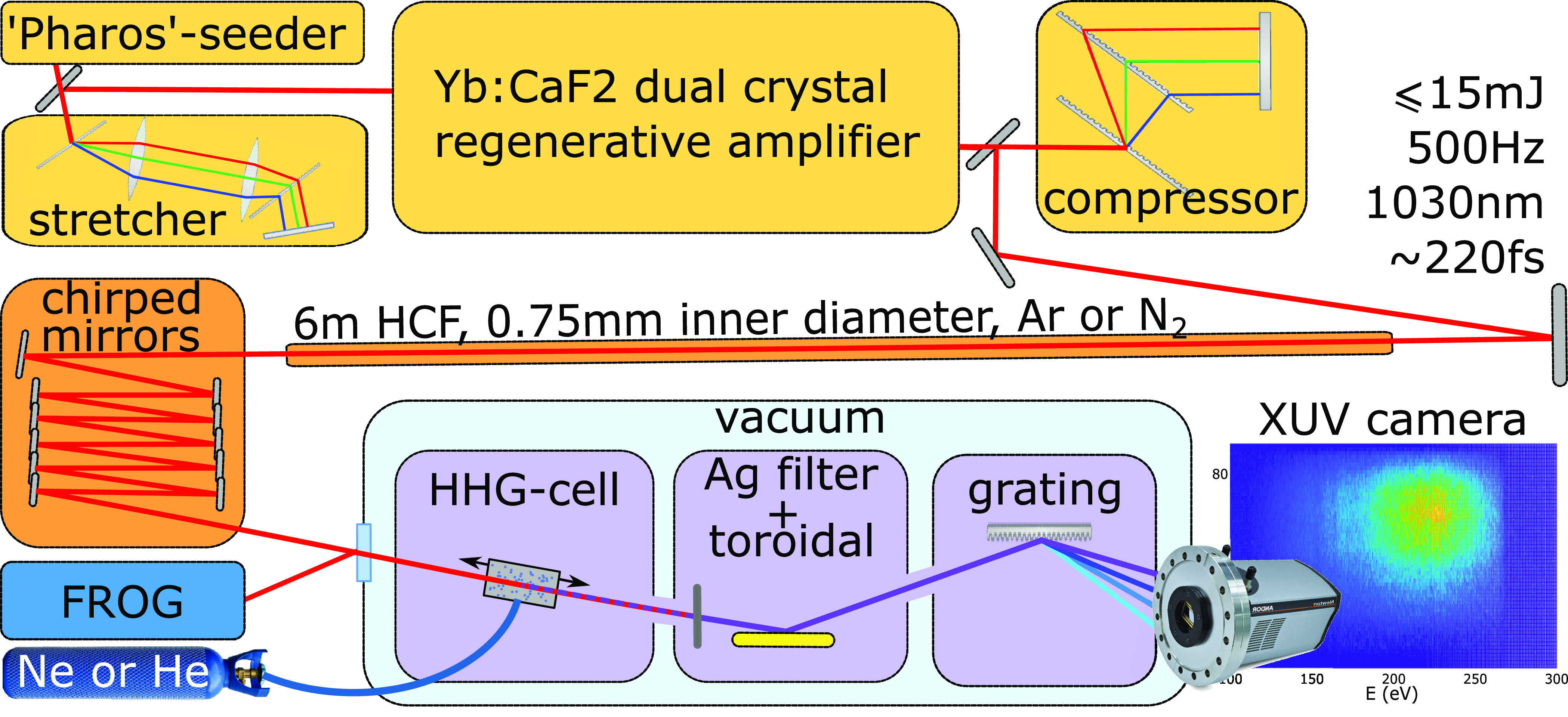
Schematic of the experimental setup. The output pulses of the laser system (up to 15 mJ, 220 fs, central wavelength 1030 nm) are coupled into a HCF (6 m long, 0.75 mm inner diameter) filled with argon or nitrogen. The output pulses from the HCF are either spectrally broadened via SPM in argon or spectrally broadened and red-shifted via SRS in nitrogen and compressed by a set of chirped mirrors. The compressed pulses enter the vacuum system of the XUV beamline through a window. The parasitic reflection of the window is used for the SHG FROG measurement.

Figure 2 shows the experimental setup. The laser system uses a chirped pulse amplification (CPA) scheme.^43^ A “Pharos” femtosecond ytterbium laser (*Light Conversion*) is used as a seeder of sub-mJ pulses that are stretched in a Martinez-type stretcher to 500 ps. Stretched pulses are then amplified in a home-built Yb:CaF_2_ cryogenically cooled dual-crystal regenerative amplifier up to 15 mJ. The amplified pulses are then compressed in a Treacy-type compressor to sub 220 fs duration. The system operates at 500 Hz repetition rate.

The laser beam is then coupled into a long, stretched HCF (*Few-cycle Inc.*, 6 m length, 0.75 mm inner diameter), where the pulses are either spectrally broadened by SPM in argon or spectrally broadened and simultaneously red-shifted by SRS in nitrogen. As discussed in the Introduction, the wavelength scaling laws for photon flux and cutoff that govern HHG dictate an optimal wavelength when targeting a specific spectral region. For generating harmonics in helium around and above the carbon K-shell absorption edge at 284 eV, based on the achieved cutoff when driving directly at 1030 nm and the scaling laws discussed in the Introduction, we can estimate this central wavelength to be between 1200 and 1300 nm. To reach this desired wavelength, the technique of red-shifting and simultaneous spectral broadening enabled through SRS by propagation in a HCF filled with molecular gas is employed.^26−28^ By adjusting the nitrogen gas pressure in the HCF the amount of red-shift Δω, which is proportional to the product of gas pressure (*p*) and laser intensity (*I*): Δω ∝ *pI*, can be continuously tuned until it is limited by the pressure-dependent critical power of self-focusing.^44^ By this the broadened spectrum reaches up to 1300 nm, with a center wavelength of 1230 nm. Afterward, a set of chirped mirrors that support a bandwidth from 650 nm up to 1350 nm (PC147 by *Ultrafast Innovations*) is used to compress the pulses to about 20 fs. Typically, we achieve such pulses by coupling 12.5 mJ pulses into the HCF with nitrogen pressure of 500 mbar. The output energy of 8 mJ is given by the combination of the coupling efficiency into the fiber (80%), the quantum losses related to the redshift (−16%) and absorption (−5%). The former parameter can, in principle, be improved by improving the beam quality after the laser amplifier, while the others are intrinsic of the redshift process.

The second harmonic generation frequency resolved optical gating (SHG FROG) setup used in our experiments was designed for pulses in excess of 20 fs. Therefore, our pulse duration measurements, the results of which are summarized in Supporting Information, Figure 1, might have overestimated the real pulse duration.^45^ The SHG FROG measurement yields pulse durations of <19 fs for SPM-compressed and <22 fs for SRS-compressed and shifted pulses. From the spectra we derive transform limited pulse durations of about 15 fs in both cases.

The laser pulses then enter a vacuum system for HHG and are focused with a *f* = 40 cm mirror into a movable gas cell of 14 mm length with a backing pressure of about 1 bar. The pulse energy can be finely tuned by closing an iris. The data shown in this paper were acquired with 4.8 mJ pulses. The pressure in the gas cell is controlled and optimized with a variable flow valve, as shown in Supporting Information, Figure 2. A 300 nm thin silver filter is used to suppress the fundamental NIR laser beam before the generated harmonics are refocused by a golden coated toroidal mirror (f = 120 cm) at 4° angle of grazing incidence on the entrance slit of a soft X-ray spectrometer (grating 001–0450 by *Hitachi* and XUV sensitive CCD camera Newton SO by *Andor*). Additional zirconium and carbon filters can be inserted to verify the measured harmonic spectra.

## Results

**Figure 3:**
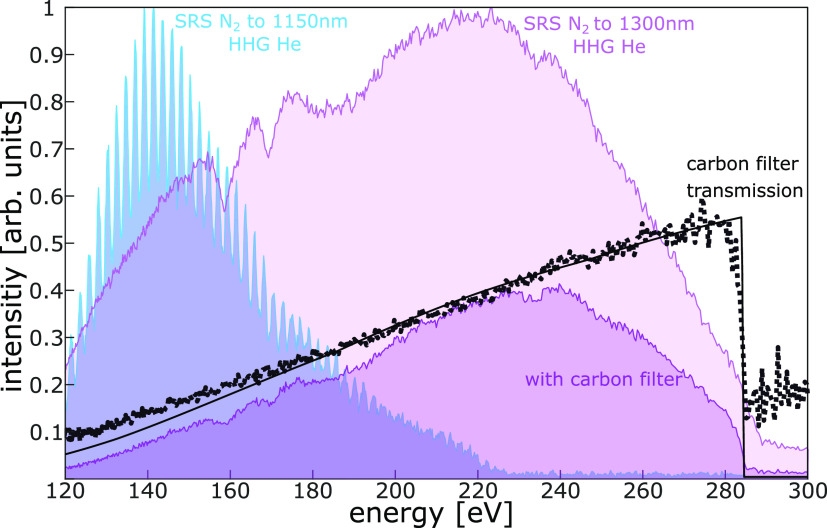
Extension of the cutoff to the carbon K-edge by increasing the redshift and bandwidth of the HHG driver. All HHG spectra are generated in helium and driven by SRS shifted and compressed pulses. Blue line, driver pulses compressed with chirped mirrors PC1611, supporting spectra up to 1150 nm. Purple lines, driver pulses compressed with chirped mirrors PC147, supporting spectra up to 1350 nm. Solid black line, theoretical transmission of the carbon filter placed before the XUV spectrometer. Black dots, ratio between the HHG spectra acquired with (dark purple) and without (light purple) carbon filter.

In our first attempts, we used the set of chirped mirrors available at the time (PC1611 by *Ultafast Innovations*), supporting a spectral bandwidth 850–1180 nm. Even though it was possible to broaden the spectrum via SRS up to 1300 nm, pulse compression was possible only for pulses with a bandwidth compatible with the chirped mirrors. With this limitation, there was no significant advantage in terms of cutoff extension when broadening the driving pulses with SRS (see blue spectrum in Figure 3) as compared to previous results with SPM.^19^ In both cases, it is not possible to exceed 220 eV. Therefore, we upgraded our setup with a set of chirped mirrors supporting a bandwidth up to 1350 nm (PC147 by *Ultafast Innovations*). HHG spectra driven by pulses with extended bandwidth and larger red-shift show a clear extension of the cutoff, as well as a more continuous structure. This is in agreement with the expectation that for shorter pulse duration and longer driving wavelengths, less laser cycles contribute to HHG.^15^

With this setting, the purple HHG spectrum shown in Figure 3 is generated and the carbon K-shell absorption edge is verified by the insertion of a thin carbon filter. In Figure 3 we show the good agreement between carbon filter transmission from literature (black)^46^ and calculation from our spectra (dashed black).

With a set of chirped mirrors fulfilling the bandwidth requirements for both SPM and SRS, it is possible to switch between the two techniques with the same experimental setup simply by filling the HCF with a noble or a molecular gas (argon and nitrogen respectively, in our experiment). The SHG FROG characterization of postcompressed pulses via SPM and SRS is shown in Supporting Information, Figure 1. The compressed pulses obtained with the two techniques are then applied for driving HHG in neon and helium.

**Figure 4:**
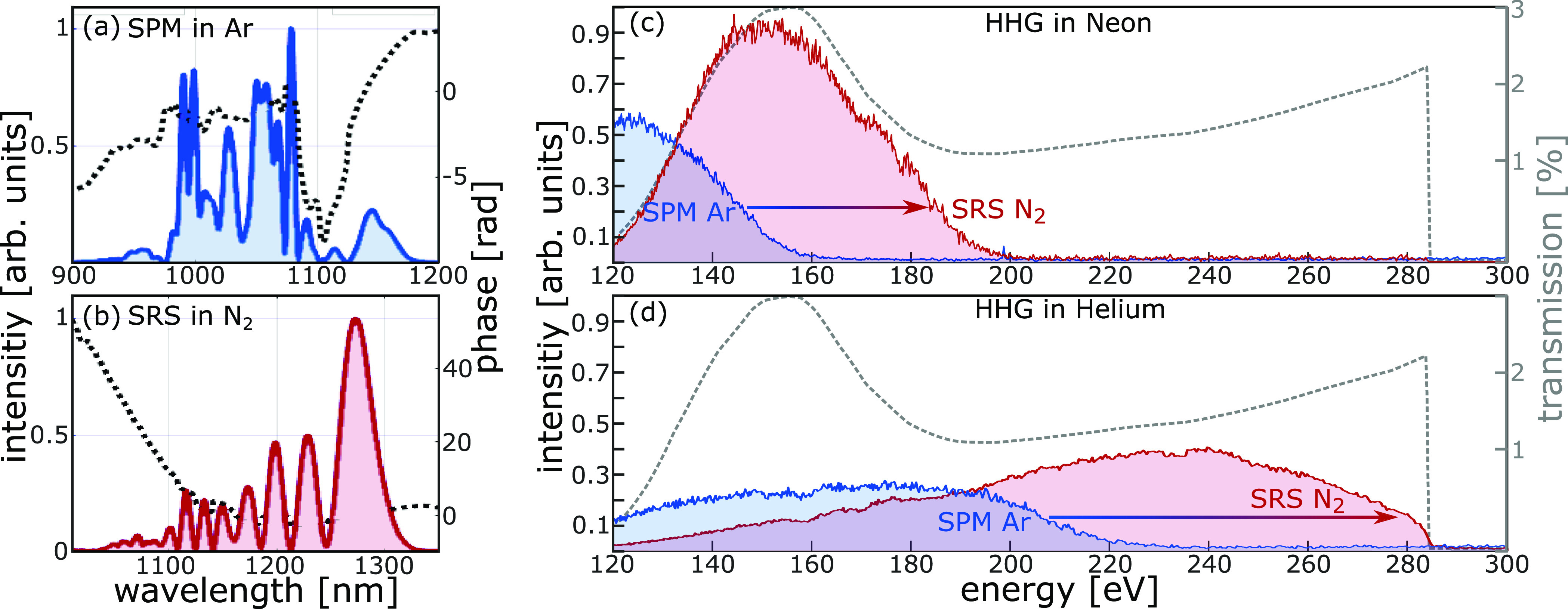
Comparison of SPM and SRS for driving HHG in neon and helium. Spectra (solid line) and phase (black dotted line) of (a) SPM-compressed and (b) SRS-compressed and shifted pulses. SHG FROG measurement yields pulse durations of <19 fs for SPM and <22 fs for the SRS case. Cutoff extension for HHG in neon (c) and helium (d) when driving with pulses from SRS in nitrogen (red) as compared to pulses from SPM in argon (blue). All XUV spectra with carbon filter and the same acquisition parameters. Dashed gray line, the transmission function of the toroidal mirror, the silver and the carbon filters.

In Figure 4, the extension of the cutoff due to the red-shift of the central wavelength of the driving pulses is clearly observable. For HHG driven in neon, the cutoff is increased from 165 to 200 eV, and for driving in helium, it is increased from 220 to 290 eV. The four spectra shown in Figure 4 are all recorded with a silver and a carbon filter and with the same acquisition parameters to be comparable among each other. The achieved flux is higher for HHG driven in neon than in helium, as expected, and the flux achieved by SPM and SRS are very similar. What may look like an increase in flux for SRS over SPM is due to the increase in transmission of the carbon filter for higher photon energies.

## Conclusion

In this work, we demonstrated the extension of the cutoff of phase-matched HHG driven by an ytterbium laser amplifier system in combination with SRS in a HCF to the carbon K-edge. To the best of our knowledge, this is the first demonstration of a driving scheme based on ytterbium lasers that is capable of reaching such photon energy without relying on OPA or OPCPA frequency down-conversion. Considering the importance of drastically increasing the photon flux at the carbon K-edge for future spectroscopic applications, there are three factors that make the proposed driving scheme particularly appealing. The first advantage concerns the laser source: Ytterbium amplifiers are particularly suitable for energy and power scaling. The second concerns the efficiency of frequency down-conversion from the laser to the HHG driver wavelength, which is higher for SRS than for OPA. Moreover, on top of the red-shift, SRS also induces enough spectral broadening to support pulse durations on the order of 20 fs. As a result, the performances in terms of delivered pulse duration can be superior to the typical scheme of Ti:Sa in combination with OPA. The third concerns the spectral range of the HHG driver. The moderate red-shift in the vicinity of the laser wavelength (1030 nm) enables to drive HHG at the carbon K-edge with the optimal wavelength (<1300 nm), which is the shortest wavelength with which the target cutoff can be still reached. As an outlook, the scheme can be further improved by slightly increasing the bandwidth and the red-shift of the driving pulses in order to upshift the maximum of the HHG spectrum. To summarize, the potential for the power scaling of the laser source and the optimized efficency for the two frequency conversion processes involved, from the laser to the NIR driver and from the NIR driver to the soft X-rays, make the proposed driving scheme the ideal platform for future developments of HHG sources in the water window, both for standard laboratories and large laser facilities.

## Supporting Information Available

Supplemental figure: Second harmonic generation frequency resolved optical gating measurements of the high harmonic generation driver pulses, HHG yield as a function of pressure (PDF)
