# Supplementary material for: HHG at the Carbon K-Edge Directly Driven by SRS Red-Shifted Pulses from an Ytterbium Amplifier
Source: ACS Photonics. 2022 Dec 29;10(1):84–91. doi: 10.1021/acsphotonics.2c01021 (PMC9853858; doi:10.1021/acsphotonics.2c01021)
Supplement: Supplementary file 1 — ph2c01021_si_001.pdf [file ph2c01021_si_001.pdf]

Supplementary information for HHG at the carbon K-edge directly driven by SRS red-shifted pulses from an ytterbium amplifier.

Martin Dorner-Kirchner,† Valentina Shumakova,†,‡ Giulio Coccia,†,¶ Edgar Kaksis,† Bruno E. Schmidt,§ Vladimir Pervak,∥,⊥ Audrius Pugzlys,†,# Andrius Baltuška,† Markus Kitzler-Zeiler,† and Paolo Antonio Carpeggiani*,†

†*Photonics Institute, Technische Universität Wien, A-1040 Vienna, Austria*

‡*Christian Doppler Laboratory for Mid-IR Spectroscopy and Semiconductor Optics, University of Vienna, A-1090, Austria*

¶*Istituto di Fotonica e Nanotecnologie-Consiglio Nazionale delle Ricerche (IFN-CNR) and Dipartimento di Fisica-Politecnico di Milano, Piazza Leonardo da Vinci 32, Milano 20133, Italy*

§*few-Cycle Inc., 1650 Blvd. Lionel Boulet, J3X 1P7, Varennes, QC, Canada*

∥*Ludwig-Maximilians-Universität München, Department of Physics, Am Coulombwall 1, 85748 Garching, Germany*

⊥*UltraFast Innovations GmbH, Am Coulombwall 1, 85748 Garching, Germany*

#*Center for Physical Sciences and Technology, Savanoriu Ave. 231, LT-02300, Vilnius, Lithuania*

E-mail: paolo.carpeggiani@tuwien.ac.at

3 pages, 2 figure

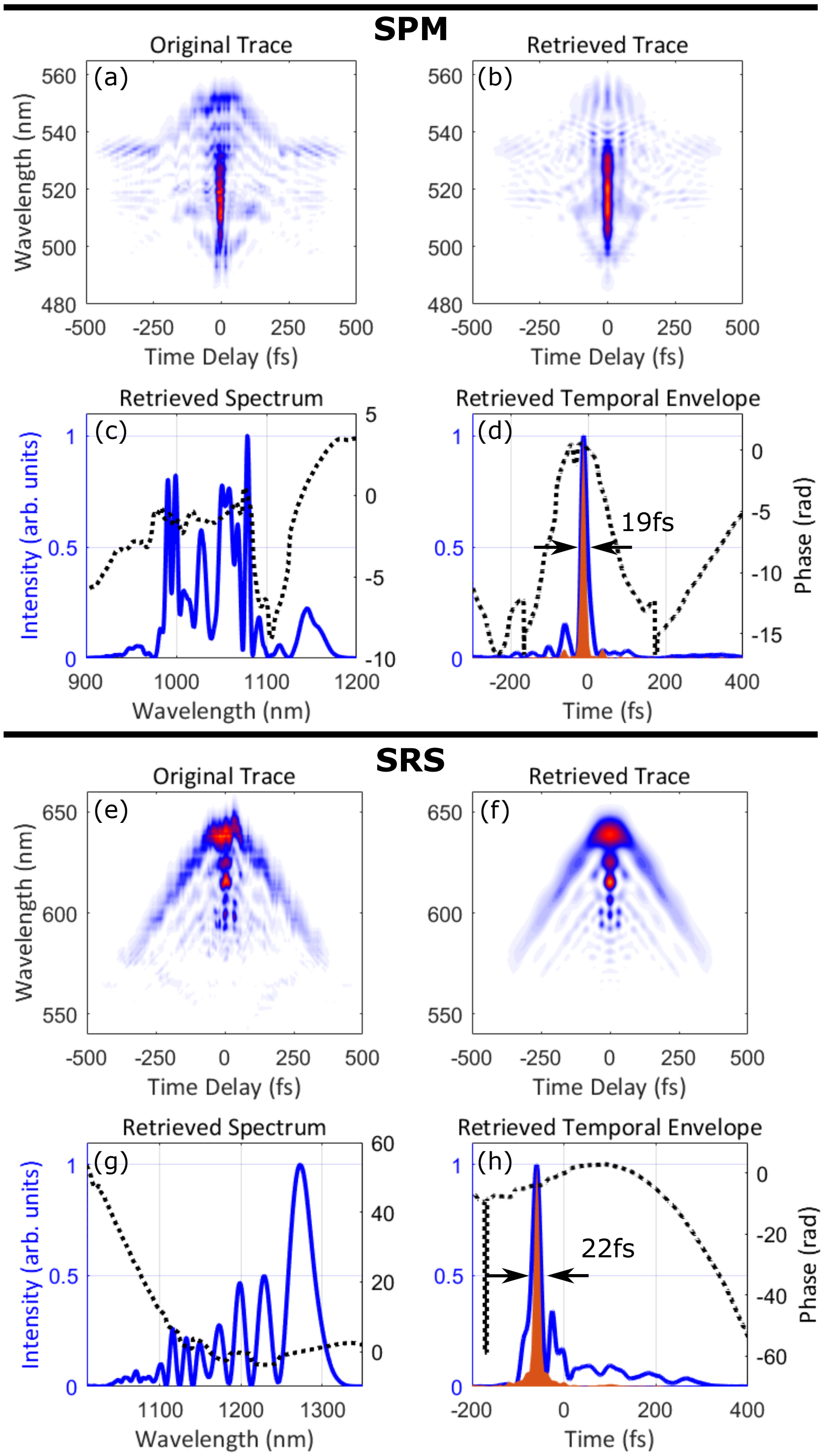

Figure S1: Second harmonic generation frequency resolved optical gating (SHG FROG) measurements of the high harmonic generation (HHG) driver pulses. Pulses compressed by chirped mirrors after self phase modulation (SPM) (top) and stimulated Raman scattering (SRS) (bottom) in the hollow core fiber (HCF). Measured (a,e) and reconstructed (b,f) SHG FROG traces. Retrieved spectrum (c,g) and temporal profile (d,h) of pulses with FWHM <19 fs (SPM) and <22 fs (SRS) used for HHG. Orange areas in (d) and (h), calculated transform limited pulses with duration of 15 fs and 14.7 fs respectively.

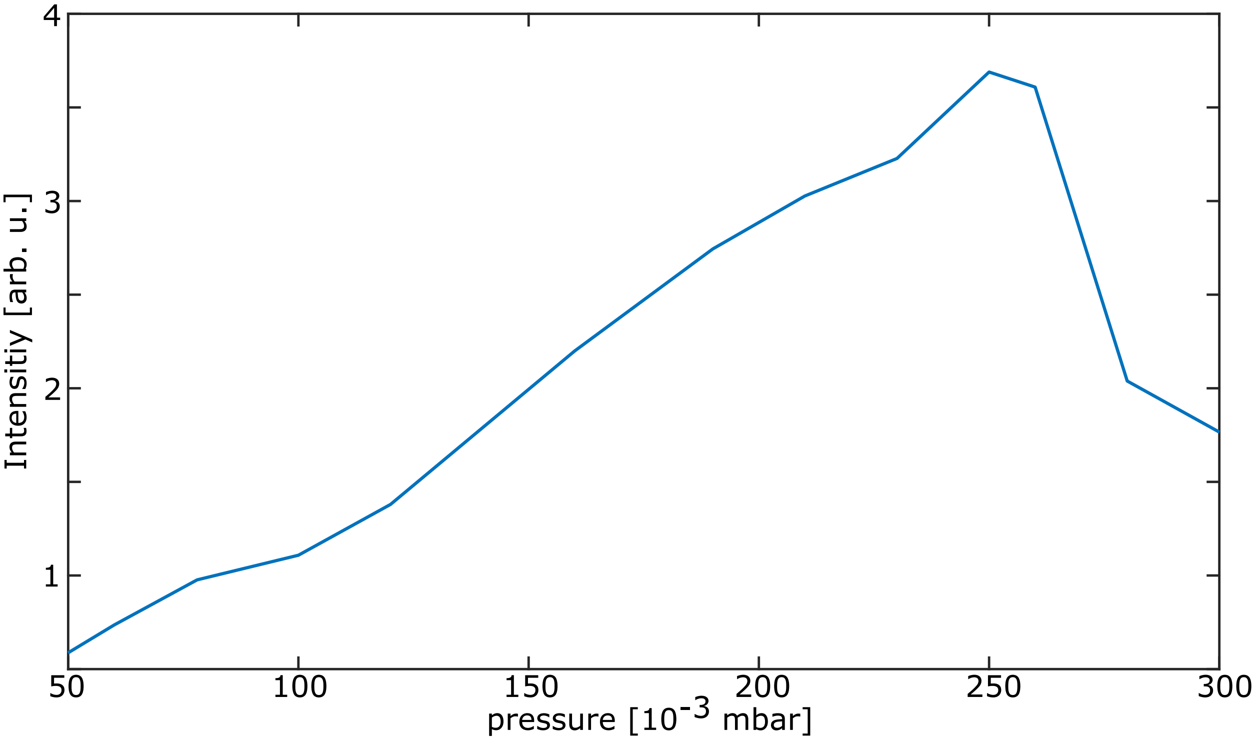

Figure S2: Extreme ultra-violet (XUV) intensity in arbitrary units for a scan of the helium gas pressure in the gas cell for HHG. The pressure in the gas cell is controlled with an analog variable flow valve with a backing pressure of about 1 bar. The pressure is not measured directly in the gas cell, but in the surrounding, continuously pumped, vacuum chamber. For each pressure step HHG parameters are optimized (pulse energy, iris, gas cell position). The XUV intensity increases with the helium gas pressure until it abruptly drops off.
